# A method for material decomposition and quantification with grating based phase CT

**DOI:** 10.1371/journal.pone.0245449

**Published:** 2021-01-22

**Authors:** Shiwo Deng, Yining Zhu, Huitao Zhang, Qian Wang, Peiping Zhu, Kai Zhang, Peng Zhang

**Affiliations:** 1 School of Mathematical Sciences, Capital Normal University, Beijing, China; 2 Beijing Advanced Innovation Center for Imaging Theory and Technology, Capital Normal University, Beijing, China; 3 Department of Electrical and Computer Engineering, University of Massachusetts Lowell, Lowell, MA, United States of America; 4 Institute of High Energy Physics, Chinese Academy of Sciences, Beijing, China; University of Notre Dame, UNITED STATES

## Abstract

Material decomposition (MD) is an important application of computer tomography (CT). For phase contrast imaging, conventional MD methods are categorized into two types with respect to different operation sequences, i.e., “before” or “after” image reconstruction. Both categories come down to two-step methods, which have the problem of noise amplification. In this study, we incorporate both phase and absorption (PA) information into MD process, and correspondingly develop a simultaneous algebraic reconstruction technique (SART). The proposed method is referred to as phase & absorption material decomposition-SART (PAMD-SART). By iteratively solving an optimization problem, material composition and substance quantification are reconstructed directly from absorption and differential phase projections. Comparing with two-step MD, the proposed one-step method is superior in noise suppression and accurate decomposition. Numerical simulations and synchrotron radiation based experiments show that PAMD-SART outperforms the classical MD method (image-based and dual-energy CT iterative method), especially for the quantitative accuracy of material equivalent atomic number.

## Introduction

Conventional polychromatic X-ray CT aims at reconstructing the linear absorption coefficient, which depends on material composition and energy-related attenuation performance. This imaging modality relies on its X-ray spectrum, which leads to a weak ability to identify substances. For example, in medical diagnosis, traditional CT often fails to distinguish iodine contrast agents and bone tissue [1]. To overcome the shortcoming, dual-energy CT is developed, which collects projections with two different X-ray spectra and is able to perform selective reconstruction for equivalent atomic number and atomic density, basic-material based concentration distributions, and so on. The commercialized dual-energy CT, like by SIEMENS and GE, can distinguish iodine contrast agents and bone tissue, tendons and ligaments, blood vessels and bone tissue, and so on [2]. The decomposition rationale for dual-energy CT depends on the fact that the energy attenuation behavior of matter varies with different X-ray spectra. However, this assumption is challenged in the real application with low-Z compounds, which have weak absorption and poor discrimination.

Different from traditional absorption-based X-ray CT, phase-based X-ray CT imaging employs both the absorption feature and the phase shift manifestation to reveal the anatomic characteristics. For low-Z material, although the absorption behavior is very similar, the performance on phase shift is quite different [3]. Therefore, theoretically, phase CT has a superior material distinguishability than absorption CT. Methodologically, the phase-contrast CT can be implemented by a grating interferometer based differential phase imaging method, which obtains three perspective images in terms of absorption, differential phase (refraction angle) and scattering. Technically, by employing CT reconstruction technique, the spatial distribution of linear absorption coefficient (***μ***), the real part of refractive index (***δ***) and the scattering coefficient can be obtained simultaneously. Assuming the X-ray is monochromatic, these physical indexes can be calculated with traditional analytical algorithms, such as filtered backprojection (FBP). Specifically, the ***μ*** and scattering coefficient can be reconstructed by the Shepp-Logan filter based FBP algorithm, while the ***δ*** can be reconstructed by the Hilbert filter based FBP [4] or BPF algorithm [5]. Moreover, they can be reconstructed from algebraic iterative reconstruction algorithm [6, 7].

Wang *et al.* employed absorption and small-angle scattering information to quantitatively measure the material composition and concentration [8], which works for the measurement of volumetric breast density (VBD) and the classification of breast micro-calcification. However, the small-angle scattering information originates from the unevenness inside a resolution unit, which is inappropriate for the substance with uniform composition inside the resolution unit but gradual variation between neighbor resolution units. To overcome this limitation and to improve the reliability of quantitative analysis, considering the high sensitivity of the phase signal, both phase and absorption information are combined for matter decomposition. In 2010, Qi *et al.* reconstructed ***μ*** and ***δ***, and meanwhile calculated the three-dimensional spatial distribution of the material electron density and the effective atomic number [9]. In 2017, Han *et al.* employed a grating interferometer to acquire absorption and differential phase information, established a two-component absorption and differential phase equation, and performed two-substrate based decomposition in projection domain [10, 11]. In 2018, Braig *et al.* proposed a two-substrate equation for material decomposition, and investigated material electron density and effective atomic number [12].

The aforementioned methods belong to a two-step strategy, which separately perform decomposition and reconstruction. One strategy is decomposing in projection domain, such as Han’s work [10, 11]. The other is in image domain, such as Qi [9] and Braig [12]. However, whatever the sequence is, noise amplification exists all the time. For projection-domain decomposition, both phase and absorption projections need to be known at the same time. When obtaining the phase projection by integrating the differential phase shift data along the X-ray refraction direction, strip artifacts are introduced and low-frequency noise are amplified in the phase projection [13, 14]. For image-domain decomposition, it is necessary to know the reconstructed ***μ*** and ***δ***. When reconstructing the ***δ***, the Hilbert filter based method lacks a noise-suppression mechanism and suffers serious noise influence. All these two approaches are presented in the Methods section.

In order to overcome the inevitable noise amplification of the two-step methods, we propose an optimization-based one-step decomposition method (PAMD-SART, phase & absorption material decomposition-SART), which directly reconstructs substrate images from the differential phase and absorption projections. Noticeably, the optimization model contains a noise-suppression mechanism and can be effectively solved by an iterative scheme.

## Theory and method

### Imaging theory

The interaction between X-rays with matter can be described by the following complex refractive index

$$\begin{array}{c} n=1-\delta +i\beta , \end{array}$$ (1)

where *δ* is the real decay rate of the refractive index, $\beta =\frac{\lambda }{4\pi }\mu$, *μ* the linear absorption coefficient, and *λ* the wavelength of the incident X-ray. When an X-ray plane wave with amplitude *A*_0_ passes through the object, the wave function of the emergent beam reads

$$\begin{array}{c} A\left(x\right)=A_{0}exp\left(i\Phi \left(x\right)\right)exp\left(-M\left(x\right)/2\right), \end{array}$$ (2)

where Φ is the matter-caused X-ray phase shift with the expression as

$$\begin{array}{c} \Phi \left(x\right)=-\frac{2\pi }{\lambda }\int_{l}\delta \left(x,y\right)dy. \end{array}$$ (3)

Let *M* describe the absorption of X-rays, which can be formularized as

$$\begin{array}{c} M\left(x\right)=\frac{4\pi }{\lambda }\int_{l}\beta \left(x,y\right)dy. \end{array}$$ (4)

After penetrating the object, the intensity of X-ray decays to

$$\begin{array}{c} I\left(x\right)={\left|A\left(x\right)\right|}^{2}=I_{0}exp(-\int_{l}\mu \left(x,y\right)dy), \end{array}$$ (5)

where $I_{0}=A_{0}\cdot A_{0}^{*}$ is the intensity of the incident X-ray, $A_{0}^{*}$ is the conjugation of *A*_0_. X-ray differential phase imaging is based on the usage of grating interferometer. By employing a phase stepping acquisition method, absorption and differential phase data can be extracted from each scan angle [15]. The absorption projection data can be written as

$$\begin{array}{c} M\left(x_{\varphi }\right)=\int \mu \left(x,y\right)dy_{\varphi }, \end{array}$$ (6)

and the differential phase projection data (refraction angle) as

$$\begin{array}{c} \theta \left(x_{\varphi }\right)=\frac{\lambda }{2\pi }\frac{\partial \Phi (x_{\varphi })}{\partial x_{\varphi }}=-\int \frac{\partial }{\partial x_{\varphi }}\delta \left(x,y\right)dy_{\varphi }. \end{array}$$ (7)

Here (*x*, *y*) is the sample coordinate system, (*x*_*φ*_, *y*_*φ*_) the measuring coordinate system, and *φ* the angle at which the measuring coordinate system rotates with respect to the sample coordinate system. In (7), ∂*δ*(*x*, *y*)/∂*x*_*φ*_ changes with the projection angle, which leads to a failure of the traditional CT algorithm. However, Hilbert filter based FBP algorithm [4] or the BPF algorithm [5] can work in this case to recover *δ*(*x*, *y*).

According to the material decomposition method, ***μ*** of the measured object can be expressed as a linear combination of ***μ*** of two basic materials. Under the condition of monochromatic X-ray exposure, ***δ*** can also be linearly expressed by ***δ*** of two basic materials [11] as follows,

$$\begin{array}{c} \left\{ \begin{array}{cc} \mu (x,y) & =f\left(x,y\right)\mu_{1}+g\left(x,y\right)\mu_{2}\\ \delta (x,y) & =f\left(x,y\right)\delta_{1}+g\left(x,y\right)\delta_{2} \end{array},\right. \end{array}$$ (8)

where *μ*_1_ and *μ*_2_ are the linear absorption coefficients of the two basic materials, *δ*_1_ and *δ*_2_ the real decay rates of the two basic materials, and *f*(*x*, *y*) and *g*(*x*, *y*) the distribution function of the two substrates. By substituting equations (6) and (7) into Eq (8) and switching the differential and integral operations, we obtain the following relationships,

$$\begin{array}{c} \left\{ \begin{array}{c} M\left(x_{\varphi }\right)=\mu_{1}(\int f\left(x,y\right)dy_{\varphi })+\mu_{2}(\int g\left(x,y\right)dy_{\varphi })\\ \theta \left(x_{\varphi }\right)=-\delta_{1}\frac{\partial }{\partial x_{\varphi }}(\int f\left(x,y\right)dy_{\varphi })-\delta_{2}\frac{\partial }{\partial x_{\varphi }}(\int g\left(x,y\right)dy_{\varphi }) \end{array}.\right. \end{array}$$ (9)

**Fig 1:**
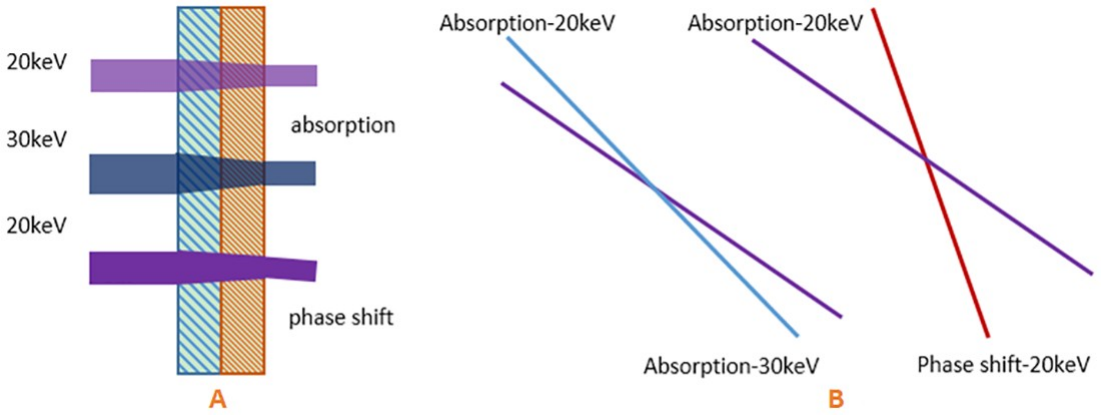
Schematic diagram. (A) Schematic diagram of ray and matter interaction and (B) the decomposition equation line.

To demonstrate the superiority of the phase and absorption based substrate decomposition than the conventional dual-energy based one, we illustrate a simple example in Fig 1. As shown in Fig 1A, assuming two X-ray beams with energy of 20 keV and 30 keV successively pass through water and PMMA with the same thickness of 1 mm, the corresponding absorption equations read,

$$\begin{array}{c} \left\{ \begin{array}{cc} \Delta_{f}\times 0.737+\Delta_{g}\times 0.623=0.136 & \\ \Delta_{f}\times 0.376+\Delta_{g}\times 0.362=0.074 \end{array},\right. \end{array}$$ (10)

where Δ_*f*_ and Δ_*g*_ are the thickness of water and PMMA, which need to be solved, and the unit of thickness is *cm*, so Δ_*f*_ = 0.1 and Δ_*g*_ = 0.1 in (10). The constant value in (10), 0.737(0.376) and 0.623(0.362) are the ***μ*** value of water and PMMA at 20(30) keV, the unit is *cm*^−1^, while 0.136(0.074) is −ln(*I*/*I*_0_) at 20(30) keV. For the 20 keV X-ray beam, according to (8), we incorporate the refractive indices and establish the following equation system,

$$\begin{array}{c} \left\{ \begin{array}{cc} \Delta_{f}\times 0.737+\Delta_{g}\times 0.623=0.136 & \\ \Delta_{f}\times 0.526+\Delta_{g}\times 0.630=0.116 \end{array},\right. \end{array}$$ (11)

where the constant values 0.526 and 0.630 in the second line of (11) are ***δ*** values of water and PMMA at 20keV, 0.116 is the calculated ***δ*** value in this configuration. As shown in Fig 1B, the three equations in (10) and (11) are graphically expressed by straight lines. The angle between the two straight lines is 3.70° for (10), and 9.93° for (11). In addition, the condition numbers of the above two equations are 36.96 and 11.66, respectively. All the evidences show that (11) is more stable and robust than (10), i.e., superior tolerance to noise and data error.

### Reconstruction algorithm

In this section, we introduce the discrete mathematical model of the phase-based basic material decomposition, investigate the derivation process, and present the implementation steps of the proposed PAMD-SART method.

#### Imaging model

Let ***f*** = (*f*_1_, *f*_2_, …*f*_*J*_)^*τ*^ and ***g*** = (*g*_1_, *g*_2_, …*g*_*J*_)^*τ*^ denote the discretized images of *f*(*x*, *y*) and *g*(*x*, *y*), where *f*_*j*_ and *g*_*j*_ are the sampled values of *f*(*x*, *y*) and *g*(*x*, *y*) at the *j*th pixel, *J* the total pixel number, and *τ* the vector transpose operation. Let $M^{\varphi }={\left(M_{1}^{\varphi },M_{2}^{\varphi },...M_{U}^{\varphi }\right)}^{\tau }$ and $\theta^{\varphi }={\left(\theta_{1}^{\varphi },\theta_{2}^{\varphi },...\theta_{U}^{\varphi }\right)}^{\tau }$ denote the absorption projection and differential phase projection extracted at projection angle *φ*, where *M*_*u*_ and *θ*_*u*_ are the extracted values by the *u*th detector cell, and *U* the number of detector cells. $A^{\varphi }={\left(a_{uj}^{\varphi }\right)}_{U\times J}$ is the projection matrix at angle *φ*, and $a_{uj}^{\varphi }$ the contribution of *f*_*j*_ and *g*_*j*_ to the *u*th ray path at projection angle *φ*. Then (9) can be discretely expressed as

$$\begin{array}{c} \left\{ \begin{array}{c} M^{\varphi }=\mu_{1}A^{\varphi }f+\mu_{2}A^{\varphi }g\\ \theta^{\varphi }=\delta_{1}D\left(A^{\varphi }f\right)+\delta_{2}D\left(A^{\varphi }g\right) \end{array},\right. \end{array}$$ (12)

where **D** is the discrete form (matrix form) of the differential operator −∂/∂*u*. Noticeably, (12) tells us that the distribution images of the two substrates can be reconstructed from differential phase and absorption projections.

#### Solution algorithm

The projection-domain based material decomposition is a traditional method [11]. This method uses the inverse of the differential operator **D**^−1^ and the boundary condition to solve **D**^−1^*θ*^*φ*^. Applying **D**^−1^ directly spreads the signal error, and leads to stripe artifacts. In order to effectively suppress these artifacts, optimization based methods are taken into consideration [13, 14]. First, (12) need to be converted as,

$$\begin{array}{c} \left\{ \begin{array}{cc} A^{\varphi }f=\frac{\delta_{2}M^{\varphi }-\mu_{2}D^{-1}\theta^{\varphi }}{\mu_{1}\delta_{2}-\mu_{2}\delta_{1}} & \\ A^{\varphi }g=\frac{\delta_{1}M^{\varphi }-\mu_{1}D^{-1}\theta^{\varphi }}{\mu_{2}\delta_{1}-\mu_{1}\delta_{2}} \end{array}.\right. \end{array}$$ (13)

Then the traditional reconstruction method is employed to recover ***f*** and ***g***. The other kind of methods can be viewed as image-domain based material decomposition, which converts (12) to

$$\begin{array}{c} \left\{ \begin{array}{c} M^{\varphi }=A^{\varphi }\left(\mu_{1}f+\mu_{2}g\right)\\ \theta^{\varphi }=D\left(A^{\varphi }\left(\delta_{1}f+\delta_{2}g\right)\right) \end{array},\right. \end{array}$$ (14)

and directly reconstruct ***μ***_1_*f* + *μ*_2_***g*** and ***δ***_1_***f*** + *δ*_2_***g***. ***μ***_1_***f*** + *μ*_2_***g*** is calculated from *M*^*φ*^ with traditional FBP algorithm, and ***δ***_1_***f*** + *δ*_2_***g*** is recovered from *θ*^*φ*^ with Hilbert filter FBP algorithm. Finally, ***f*** and ***g*** are easily obtained by solving an linear equation system with two variables. Apparently, the first method contains an integral operation in the projection domain, and the second method requires a Hilbert filter operation in the image domain, both of which inevitably amplify the noise. If we use an optimization based reconstruction algorithm to recover ***μ*** [6] or ***δ*** [7], the noise can be suppressed. However, the edge of low contrast object might be blurred, since it is difficult to adjust the regularization parameters to balance the noise and the edge in a image with high dynamic range.

#### PAMD-SART algorithm

The algorithm proposed in this paper is to obtain the final decomposition image by solving the following optimization model:

$$\begin{array}{c} \begin{array}{cc} (f^{*},g^{*})= & \arg\min_{f,g}\{\sum_{\varphi =1}^{\pi }({∥M^{\varphi }-\left(\mu_{1}A^{\varphi }f+\mu_{2}A^{\varphi }g\right)∥}^{2}+\\ & {∥\theta^{\varphi }-\left(\delta_{1}D\left(A^{\varphi }f\right)+\delta_{2}D\left(A^{\varphi }g\right)\right)∥}^{2})+R\left(f\right)+R\left(g\right)\}. \end{array} \end{array}$$ (15)

The definition of each symbol is consistent with section Imaging model and **R**(⋅) is a regularization operator such as total variation (TV) [16, 17], gradient L0 [18], Mumford-Shah [19] or an image filter. In the following, inspired by the SART reconstruction strategy [20], an iterative algorithm to directly reconstruct ***f*** and ***g*** from the absorption projection *M*^*φ*^ and the differential phase projection *θ*^*φ*^ is given.

Assuming the current estimate images are (***f***^(*m*)^, ***g***^(*m*)^), then the projection estimations of absorption and differential phase can be calculated by

$$\begin{array}{c} \left\{ \begin{array}{c} M^{\varphi (m)}=\mu_{1}A^{\varphi }f^{\left(m\right)}+\mu_{2}A^{\varphi }g^{\left(m\right)}\\ \theta^{\varphi (m)}=\delta_{1}D\left(A^{\varphi }f^{\left(m\right)}\right)+\delta_{2}D\left(A^{\varphi }g^{\left(m\right)}\right) \end{array}.\right. \end{array}$$ (16)

Furthermore, the residual can be expressed as $e_{M}^{\left(m\right)}=M^{\varphi }-M^{\varphi (m)}$ and $e_{D^{-1}\theta }^{\left(m\right)}=D^{-1}\left(\theta^{\varphi }-\theta^{\varphi (m)}\right)$. By simplifying (15) to each angle and setting the absorption and phase term to zero, one can get the follow equations,

$$\begin{array}{c} \left\{ \begin{array}{c} \mu_{1}A^{\varphi }\left(f-f^{\left(m\right)}\right)+\mu_{2}A^{\varphi }\left(f-f^{\left(m\right)}\right)=e_{M}^{\left(m\right)}\\ \delta_{1}A^{\varphi }\left(g-g^{\left(m\right)}\right)+\delta_{2}A^{\varphi }\left(g-g^{\left(m\right)}\right)=e_{D^{-1}\theta }^{\left(m\right)} \end{array}.\right. \end{array}$$ (17)

After solving the above equations, we obtain

$$\begin{array}{c} \left\{ \begin{array}{c} A^{\varphi }\left(f-f^{\left(m\right)}\right)=\frac{\delta_{2}e_{M}^{\left(m\right)}-\mu_{2}e_{D^{-1}\theta }^{\left(m\right)}}{\mu_{1}\delta_{2}-\mu_{2}\delta_{1}}\\ A^{\varphi }\left(g-g^{\left(m\right)}\right)=\frac{\delta_{1}e_{M}^{\left(m\right)}-\mu_{1}e_{D^{-1}\theta }^{\left(m\right)}}{\mu_{2}\delta_{1}-\mu_{1}\delta_{2}} \end{array}.\right. \end{array}$$ (18)

Finally, by using SART algorithm, we get the iterative form

$$\begin{array}{c} \left\{ \begin{array}{c} f_{j}^{\left(m+1\right)}=f_{j}^{\left(m\right)}+\frac{\lambda }{A_{+,j}^{\varphi }}\sum_{u=1}^{U}\frac{a_{u,j}^{\varphi }}{A_{u,+}^{\varphi }}(\frac{\delta_{2}e_{M,u}^{\left(m\right)}-\mu_{2}e_{D^{-1}\theta ,u}^{\left(m\right)}}{\mu_{1}\delta_{2}-\mu_{2}\delta_{1}})\\ g_{j}^{\left(m+1\right)}=g_{j}^{\left(m\right)}+\frac{\lambda }{A_{+,j}^{\varphi }}\sum_{u=1}^{U}\frac{a_{u,j}^{\varphi }}{A_{u,+}^{\varphi }}(\frac{\delta_{1}e_{M,u}^{\left(m\right)}-\mu_{1}e_{D^{-1}\theta }^{\left(m\right)}}{\mu_{2}\delta_{1}-\mu_{1}\delta_{2}}) \end{array},\right. \end{array}$$ (19)

where $e_{M,u}^{\left(m\right)}$ and $e_{D^{-1}\theta ,u}^{\left(m\right)}$ are the *u*th components of $e_{M}^{\left(m\right)}$ and $e_{D^{-1}\theta }^{\left(m\right)}$, *λ* the relaxation factor, $A_{u,+}^{\varphi }=\sum_{j=1}^{J}a_{u,j}^{\varphi }$ and $A_{+,j}^{\varphi }=\sum_{u=1}^{U}a_{u,j}^{\varphi }$ with *u* = 1, 2, …*U* and *j* = 1, 2, …*J*.

In the iterative scheme, the calculation of phase residual $e_{D^{-1}\theta }^{\left(m\right)}$ requires to inverse the differential operator **D**^−1^, which may diffuse the error. In order to overcome the shortcoming, we employ the following optimization model

$$\begin{array}{c} e_{D^{-1}\theta }^{(m)*}=arg\min_{e}{∥D\left(e\right)-\left(\theta^{\varphi }-\theta^{\varphi (m)}\right)∥}_{2}^{2}, \end{array}$$ (20)

and use a gradient descent method with a fixed step number in real applications. The elaborated iteration steps of the PAMD-SART method is shown in Algorithm 1.

**Algorithm 1 The PAMD-SART Method**

1: Initialization: ***f***^(0)^ = **0**, ***g***^(0)^ = **0**, *m* = 0

2: Calculate the estimate of absorption projection *M*^*φ*(*m*)^ and its residuals $e_{M}^{\left(m\right)}$; Calculate the differential phase projection estimate *θ*^*φ*(*m*)^, use (20) to calculate the residuals $e_{D^{-1}\theta }^{\left(m\right)}$

3: Iteratively solve ***f***^(*m*+ 1)^, ***g***^(*m*+1)^ according to (19).

4: Do a regularization operator to ***f***^(*m*+1)^, ***g***^(*m*+1)^.

5: Set *m* = *m* + 1 and turn to step (2) until the stop condition is met.

6: Return ***f***^(*m*)^, ***g***^(*m*)^

## Verification

In this section, we performed numerical simulations and real experiments to verify the proposed method, including visual comparison of material decomposition results and quantitative analysis of acquired equivalent atomic number.

The image quality was measured with peak signal-tonoise ratio (PSNR)

$$\begin{array}{c} PSNR=10log_{10}(\frac{N\cdot Gr^{2}}{{∥I-I^{*}∥}_{2}^{2}}), \end{array}$$ (21)

where *I* is a reconstructed image, I* the ground truth image, *N* the number of image pixels in *I*, and *Gr* the maximum gray value of I*.

### Numerical simulations

**Fig 2:**
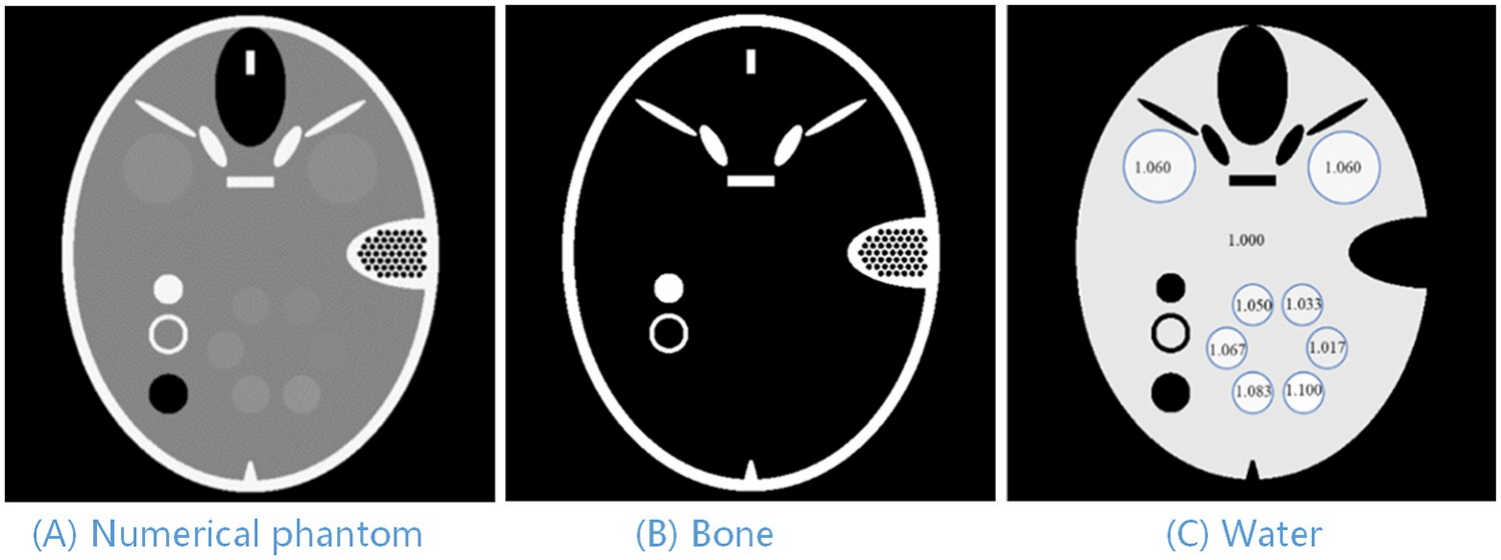
Phantom in numerical experiments. (A) Numerical phantom, (B) The Bone material of the phantom, and (C) The Water material of the phantom.

The numerical phantom was derived from the FORBILD head phantom [21]. As shown in Fig 2A, the size is 9.8mm*7.8mm, including bone and water. In order to enhance the comparison performance, we added multiple water-like objects to the original image. The specific density is shown in Fig 2C. In the simulated experiment, for the sake of simplicity, we used 20 keV monochromatic X-ray. The *μ* and *δ* of water and bone were from the X-ray optics software toolkit(XOP) [22]. First, the projection data of the sample was obtained using the forward projection method, and then Poisson noise was added, where the number of original photons was 10^6^ per ray. The differential phase projection was obtained by performing center-difference to the phase projection, and the *δ* values of water and bone were multiplied by the same ratio so that they were similar to the absorption value *μ*. In the simulation, a parallel-beam setting was used to acquire 540 projections equally spaced in 180 degrees. The detector contained 512 cells with a size of 20 um. The diameter of the covered field of view was 10.24 mm.

In image reconstruction, bone and water were selected as the basic materials, and the reconstructed image size was 512 × 512. The PAMD-SART method was implemented by C++ and CUDA with GPU acceleration. For better comparison, we implemented regularized iterative method to reconstruct ***μ*** [6] and ***δ*** [7] in the first step of the image based method. TV was employed as a regularization term and the solution algorithm was from the Chambolle’s method [17]. This method is very efficient and easy to be implemented, of which only one regularization parameter (*λ*_*TV*_) is used to adjust the image fidelity and TV. According to our experimental results, *λ*_*TV*_ = 0.1*mean(*image*) was a good beginning for Chambolle’s method. Specifically, ray-casting method was used for forward projection process, and pixel-driven method for backward projection.

**Fig 3:**
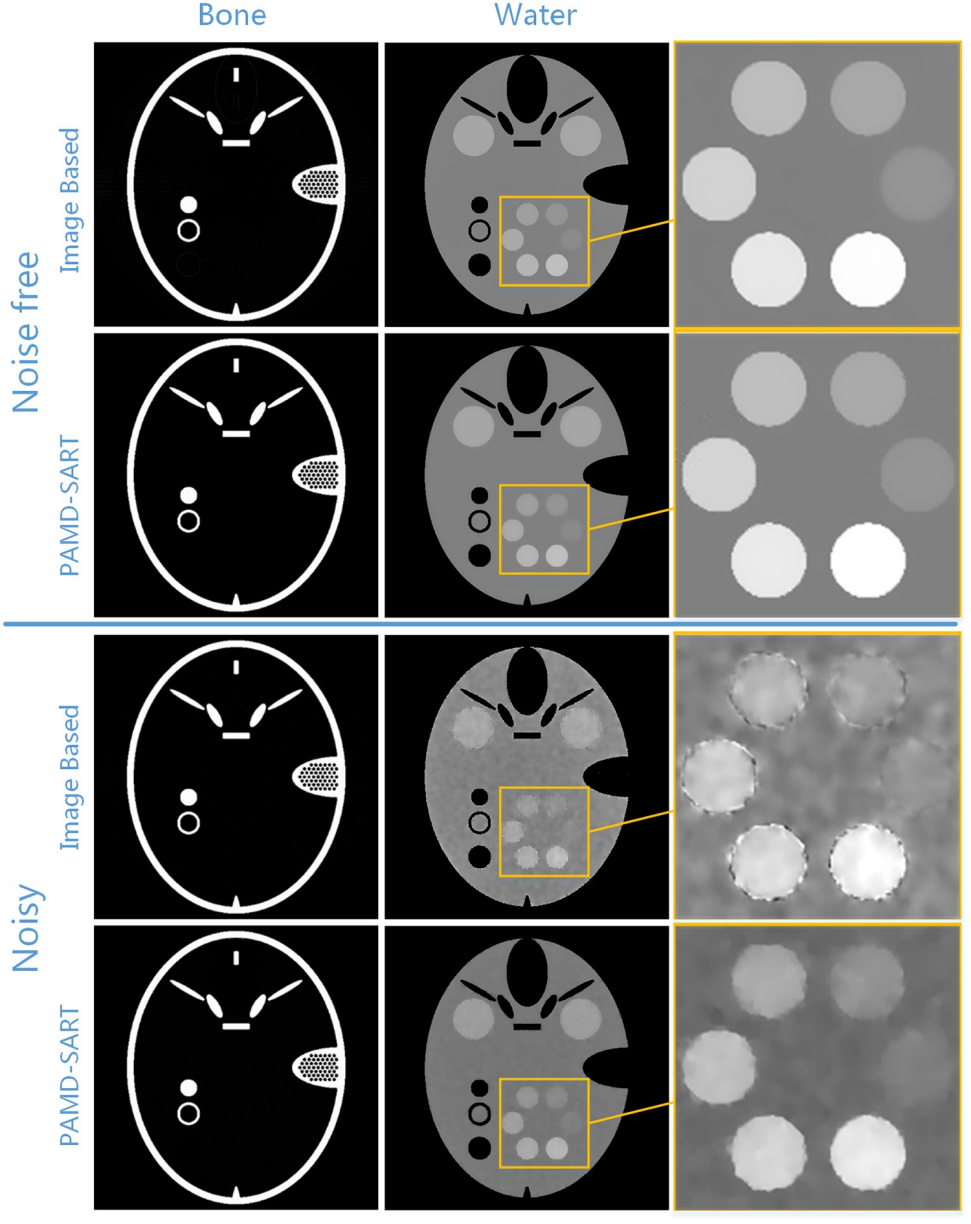
Reconstruction result of phantom study. Comparison of image-based and PAMD-SART results in noise free and noisy case. All the bone fraction images are displayed in gray window [0, 1], and water in [0.8, 1.2].

**Fig 4:**
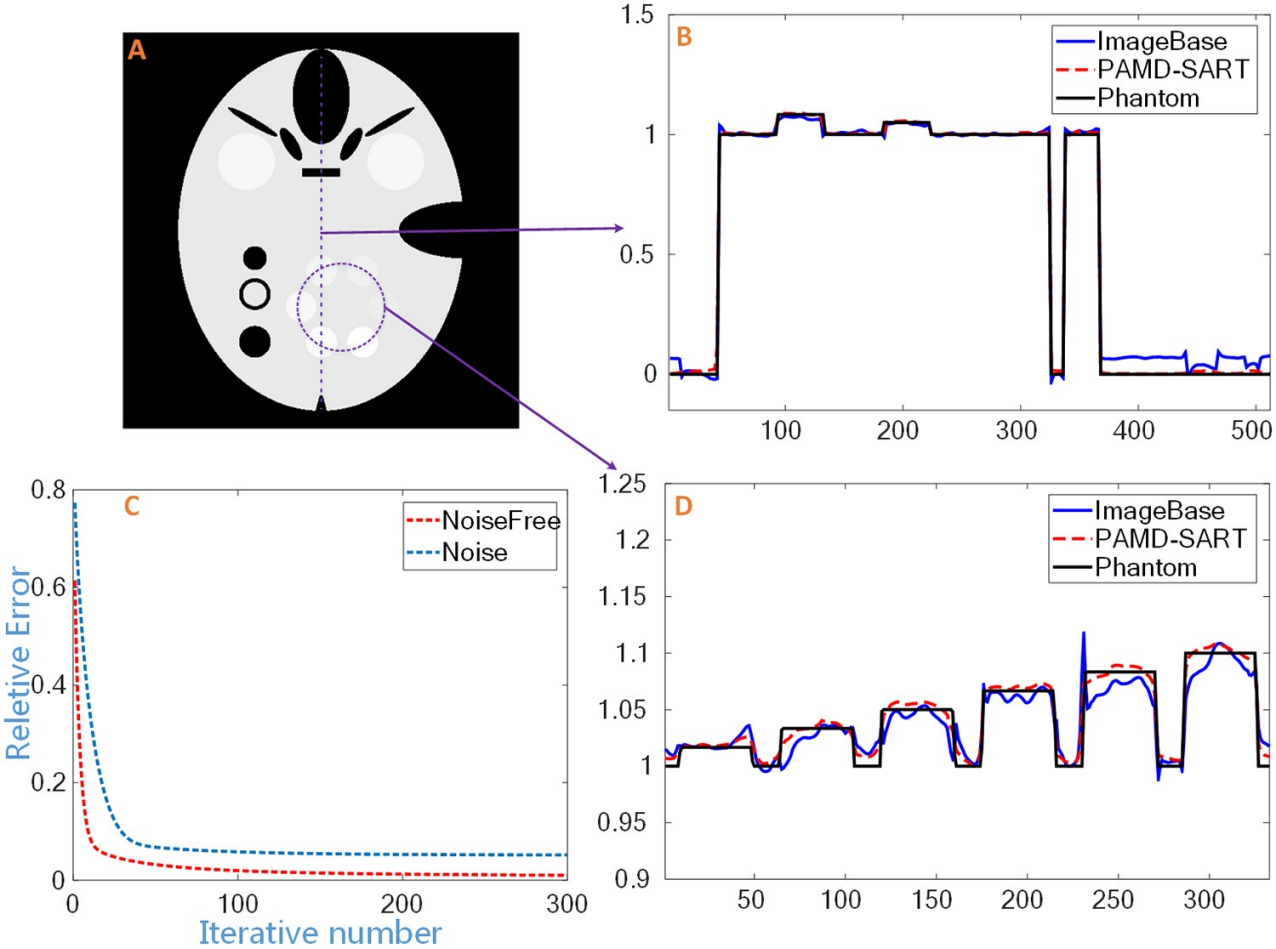
Profile of water-based reconstruction image in noisy case. The vertical (B) and circular (D) profile in the water-based image (A), and (C) the convergence curve of the PAMD-SART method.

The decomposed results under noise-free and noisy cases are shown in Fig 3. For each case, both image-based method and PAMD-SART were performed. We zoomed in the local region of water-like objects and illustrated in the third column to improve the visual comparison. The profile of water-based image in noisy case and the convergence curve of the PAMD-SART method are shown in Fig 4. The PSNR values for both methods in the noise-free and noise cases are shown in Table 1.

**Table 1:** PSNR comparison of decomposition results.

	Noise free		Noise	
	Image-Based	PAMD-SART	Image-Based	PAMD-SART
bone	51.47	55.06	32.31	36.01
water	40.66	43.70	26.69	32.02

From the results in Figs 3 and 4 and Table 1, we can see that the PAMD-SART method is superior to the image-based method. In the noise-free case, looking at the first and second lines of Fig 3, both methods can reconstruct the result without artifacts, but when comparing the circle structure with the lowest contrast in the third column, the boundary of the structure in image-based method is a little blurry, while the result of PAMD-SART is very sharp. In the noisy case, PAMD-SART performs much better than image-based method. From Table 1, the PSNR value of the image-based method is smaller than PAMD-SART, which indicates that the former keeps a larger error. From the circular water-like material region in the third column of Fig 3, the two materials with the lowest density can hardly be distinguished from the image-based method, but are clear in PAMD-SART. From the profile in Fig 4, we can see that there is a large oscillation in the image-based result, and the material with smallest density is too close to the water to be separated. However, PAMD-SART can well identify the small contrast change. The curve of relative error indicates the convergence of PMAD-SART in numerical.

The reason for this result is that the original image has a large dynamic range, it is difficult to balance the fidelity term and the regularization term when directly regularizing the original image. When suppressing noise artifacts, the border of low contrast objects will inevitably be blurred. The proposed PAMD-SART method uses projection information of ***μ*** and ***δ*** simultaneously in the reconstruction process, which reduces the noise interference. Moreover, PAMD-SART performs the regularization on substrate images which has smaller dynamic range than the original images, so has less effect on low-contrast objects. Because PAMD-SART has the advantages in these two aspects, it is much superior to the image-based method.

### Real experiment

Real experiments were performed using the X-ray grating interferometer on the BL13W experimental station by Shanghai Synchrotron Radiation Facility. The grating interferometer consisted of a *π*/2 phase grating with a period of 2.396 um and an absorption grating with a period of 2.4 um. The distance between the two gratings was 46.38 mm and the photon energy used in the experiment was 20 keV. The sCMOS X-ray detector with effective pixel array of 2048 × 600 was applied, and the pixel size was 6.5 × 6.5 *um*^2^. In order to obtain the phase and absorption projection, the step method was used for data acquisition. The number of steps was 8, which meant the phase grating moved 8 steps in a period to take samples separately, then completed the data acquisition from one angle. Finally, the data of 540 angles were collected at equal intervals within 180 degrees. The average digital value of 8 referenced acquired image in a period was about 25000, and the fringe visibility about 50%. The absorption and differential phase projections were calculated from the data collected at each angle by the phase step formula in [23]. The reconstructed image size was 1024 × 1024, and the maximum iterations was set as 200 which was the stop condition of PAMD-SART.

#### Phantom experiment

**Fig 5:**
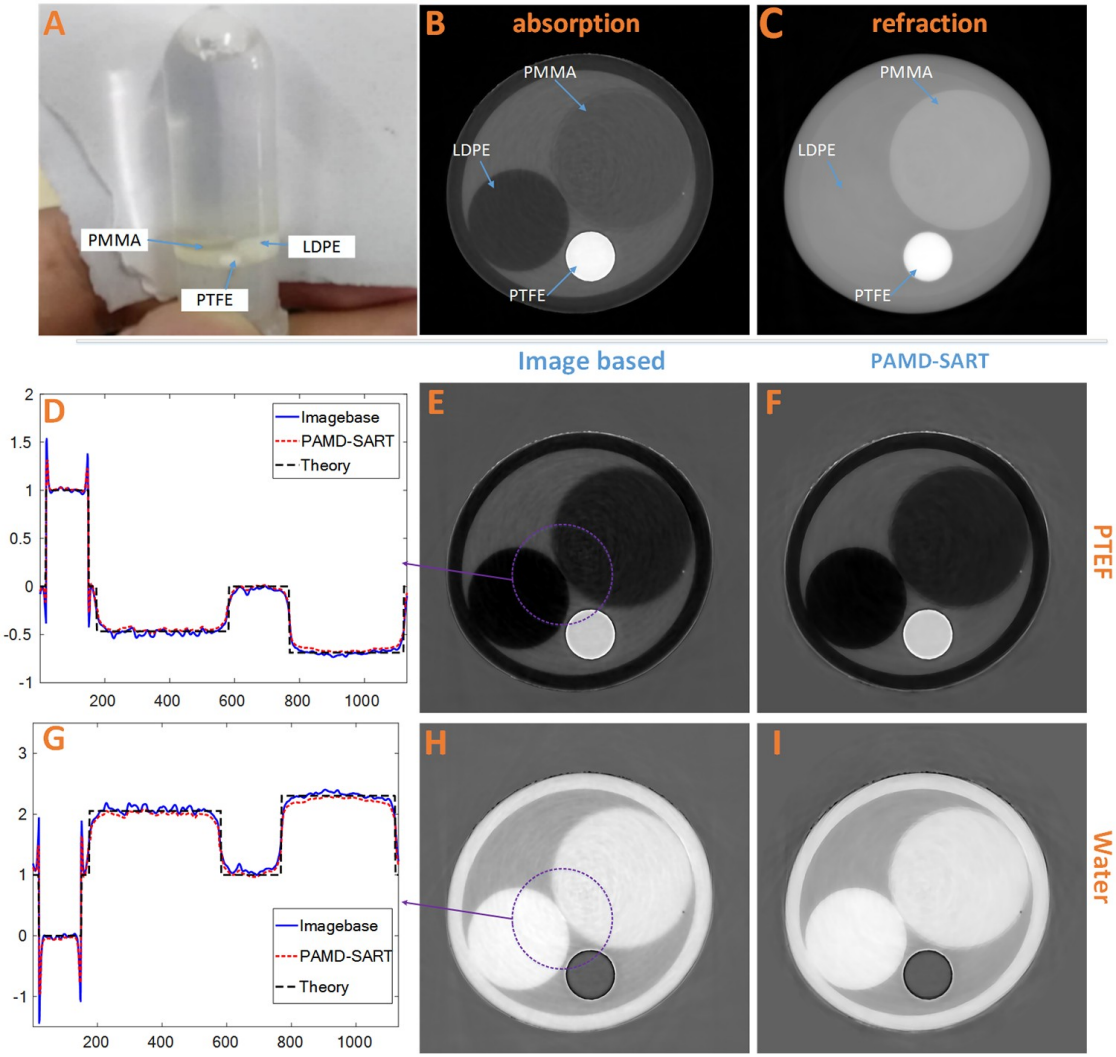
Experimental results of phantoms. (A) The physical photo, (B) the reconstructed tomogram of the ***μ*** and (C) the ***δ***; the second and third lines are the PTFE-based and water-based results decomposed by the image-base method and the PAMD-SART method, including the line Comparison chart and tomogram. (D) is the profile line of PTFE-based result, while (G) the water-based result; (E) and (F) are PTFE-based decomposed result of method image based and PAMD-SART; (H) and (i) are water-based decomposed result of method image based and PAMD-SART. The display window for B is [0, 0.2], C [0, 8.2], E and F [-0.7, 1.4], H and I [-1, 2.5].

As shown in Fig 5A, the phantom consists of four components, Low Density Polyethylene (LDPE), Polymethyl Methacrylate (PMMA), Polytetrafluoroethylene (PTFE), and water. The PTFE, LDPE and PMMA cylinders with diameters of 2.0 mm, 4.0 mm and 5.6 mm, respectively, were placed in a polyethylene plastic tube with an external diameter of 10.7 mm and injected with pure water to form the whole sample.

In this experiment, we used water and PTFE as substrates to perform PAMD-SART and image-based decomposition. Then based on the substrate images, ***μ*** and ***δ*** images and equivalent atomic number were calculated. This result was compared with the dual energy CT method.

Fig 5 shows the decomposition results of different methods. Fig 5B is the absorption coefficient tomography. The outer ring in the image represents the polyethylene plastic container, and there are three different components in the inner ring, namely, three circles with different gray levels in the middle of the ring, LDPE phantom with the lowest gray level on the left, PTFE phantom with the highest gray level at the bottom, PMMA phantom at the upper right and water at the rest. In Fig 5C, it can be seen that LDPE and water are difficult to be distinguished. This phenomenon shows that the contrast of phase information is not necessarily higher than the absorption. Comparing the decomposition results of image-based and PAMD-SART methods, i.e., Fig 5D–5I, it is obvious that PAMD-SART method has better noise removal performance.

Both works of Qi [9] and Braig [12], refer to the calculation of electron density *ρ*_*e*_ and equivalent atomic number *Z* from absorption *μ* and phase *δ*, by employing the following relationship

$$\begin{array}{c} \left\{ \begin{array}{cc} \mu (E,x,y) & =C_{p}/E^{C_{E}}\rho_{e}\left(x,y\right)Z{\left(x,y\right)}^{C_{Z}}+C_{KN}\left(E\right)\rho_{e}\left(x,y\right)\\ \delta (E,x,y) & =C_{PC}\left(E\right)\rho_{e}\left(x,y\right) \end{array},\right. \end{array}$$ (22)

where *C*_*p*_, *C*_*E*_, *C*_*Z*_ are parameters to be determined, $C_{PC}\left(E\right)=\frac{r_{0}h^{2}c^{2}}{2\pi E^{2}}$, *r*_0_ the classical radius of the electron, *h* the Planck constant, *c* the speed of light, and the Klein-Nishina cross section as follow,

$$\begin{array}{c} \begin{array}{c} C_{KN}\left(E\right)=2\pi r_{0}^{2}(\frac{1+a}{a^{2}}\left(\frac{2(1+a)}{1+2a}-\frac{ln(1+2a)}{a}\right)+\frac{ln(1+2a)}{2a}-\frac{1+3a}{{\left(1+2a\right)}^{2}}) \end{array} \end{array}$$ (23)

with *a* = *E*/511 *keV* the relative mass energy to electron. After obtaining *ρ*_*e*_ and *Z*, the virtual absorption coefficient tomogram at other energies can be further calculated.

In the proposed method, by using the obtained substrate images, the refraction and absorption images can be calculated by (8), and then introduced to (22) to get *ρ*_*e*_ and *Z*. The theoretical equivalent atomic number *Z* for a compound was calculated by the following equation [24]

$$\begin{array}{c} Z={\left(\sum_{j}\omega_{j}Z_{j}^{2.94}\right)}^{1/2.94}, \end{array}$$ (24)

where *ω*_*j*_ is the fraction of the total number of electrons associated with each element, and *Z*_*j*_ is the atomic number of each element. According to the water absorption coefficient relationship with energy, the calculated equivalent energy is 20.22 Kev. The absorption and phase coefficient of the four materials (water, PMMA, PTFE and LDPE) are used in (22) to fit the coefficient in the ***μ*** formula. After fitting, the coefficients are *C*_*p*_ = 1.004, *C*_*E*_ = 3.027, and *C*_*Z*_ = 3.619.

Using the ***μ*** in formula (22) with two different energies, the atomic number can also be calculated, which is employed in dual-energy CT [25]. Hence, we acquired two CT datasets by using 20keV and 12keV on the synchrotron radiation station, and the number of views was the same as the grating based scanning. The integration time of detector was adjusted, so that the digital value of acquired image under direct exposure was around 50000. Compared to the data acquired with grating, from the view of detector, the total dose of two monochromatic CT was about half of the grating.

**Fig 6:**
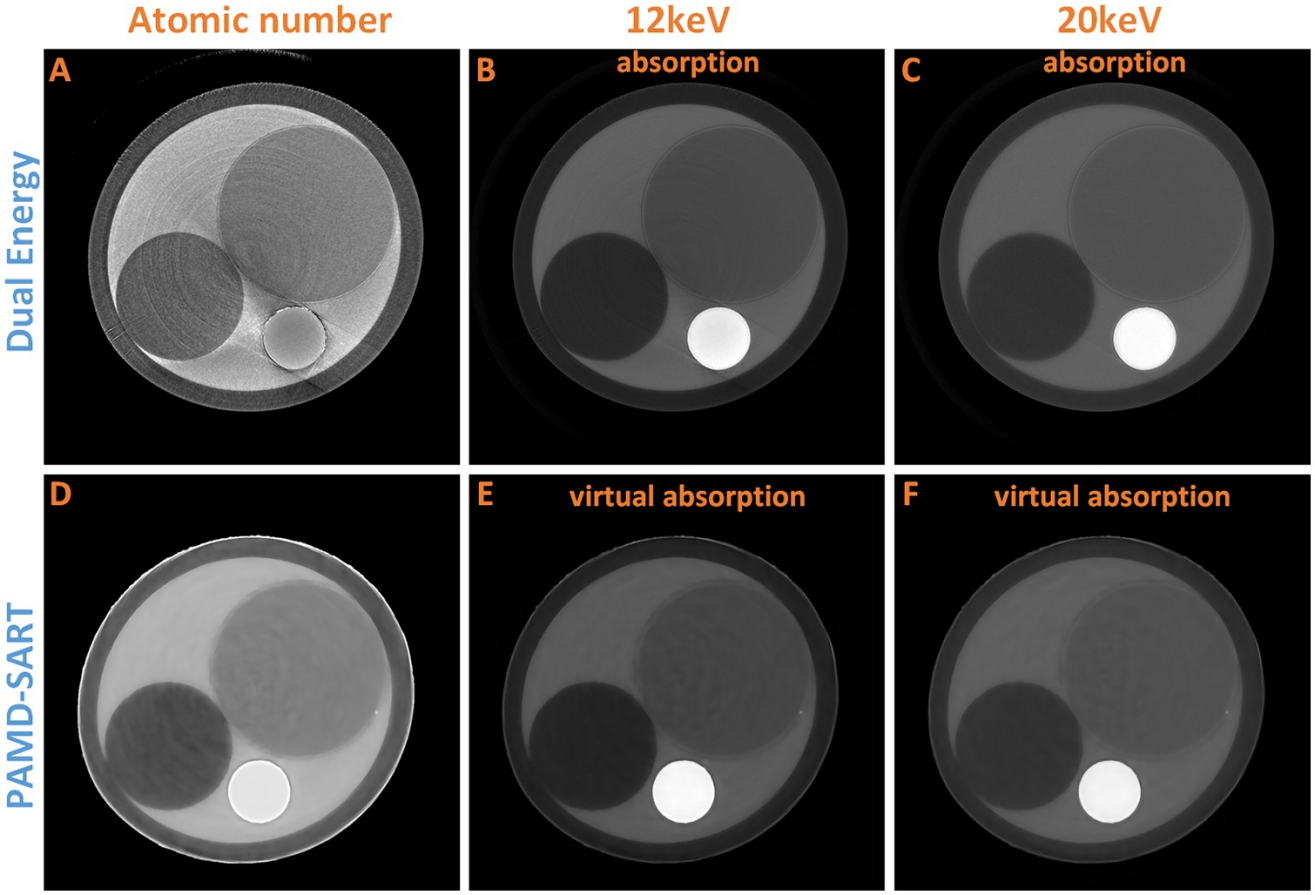
Phantom results of atomic number. (A) is the equivalent atomic number by dual energy method using absorption of 12keV (B) and 20keV (C). (D) is the equivalent atomic number by PAMD-SART method. (E) and (F) are the virtual absorption at 12keV and 20keV respectively. The display window for A and D are [5,9], B [0,0.7], C and F [0,0.2], and E [0,0.8].

As shown in Table 2, the fitted results are very close to the theoretical values. Fig 6 and Table 2 show that the accuracy of the atomic tomographic image reconstructed by the substrate image is close to the theoretical value, while the result by the dual-energy method contains a large error for PTFE and LDPE. Theoretically, the equivalent atomic number of PTFE should be greater than water. However, when using the dual-energy method with 12 keV and 20 keV X-ray beams, the calculated atomic number of PTFE is smaller than water. Once *ρ*_*e*_(*x*, *y*) and *Z*(*x*, *y*) were obtained, ***μ***(*x*, *y*) at any energy can be calculated by formula 22, which generated a virtual image. In the 12 keV virtual image, it can be found that the ***μ*** ratio of PTFE to water is 2.78, while is 2.41 in the 12 keV actual absorption image. These results demonstrate that the empirical formula of atomic number calculated by dual energy (*Z*_*C*_(*μ*_1_, *μ*_2_)) has a large error in the low energy case. Furthermore, by comparing and analyzing the absorption coefficients of various substances, it can be found that the empirical formula (*Z*_*C*_(*μ*_1_, *μ*_2_)) is no longer valid when the X-ray energy is less than 20 keV. This is mainly because the linear attenuation coefficient in (22) ignores the coherent scattering term (Rayleigh cross section). However, Table 3 shows that in the energy below 30 keV this term is non-negligible. In Table 3, the cross section of photon interaction are from xraylib [26], these data can also be found in Evaluated Nuclear Data Library(ENDL) [27].

**Table 2:** Phase, absorption information and equivalent atomic number.

	Water	PTFE	PMMA	LDPE
*δ*(20keV)	5.653e-7	1.039e-6	6.777e-7	5.863e-7
*μ*_1_(20keV)	7.369e-1	1.907	6.280e-1	3.905e-1
*μ*_2_(12keV)	2.729	6.776	2.111	1.195
*Z*_*T*_	7.417	8.433	6.467	5.444
*Z*_*C*_(*δ*, *μ*_1_)	7.416±0.10	8.398±0.04	6.433±0.11	5.409±0.12
*Z*_*C*_(*μ*_1_, *μ*_2_)	7.410±0.24	6.881±0.15	6.612±0.15	6.002±0.18

(*Z*_*T*_ is the theoretical value by (24), *Z*_*C*_ is the calculated value).

**Table 3:** Cross section of water and PTFE.

*cm*^2^/*g*	Water			PTFE		
	12 keV	20 keV	30 keV	12 keV	20 keV	30 keV
Photoionzation	2.7828	0.5438	0.1457	3.6262	0.7170	0.1938
Compton	0.1625	0.1774	0.1829	0.1305	0.1473	0.1544
Rayleigh	0.1805	0.0885	0.0469	0.2117	0.1025	0.0544

#### Biological sample experiment

**Fig 7:**
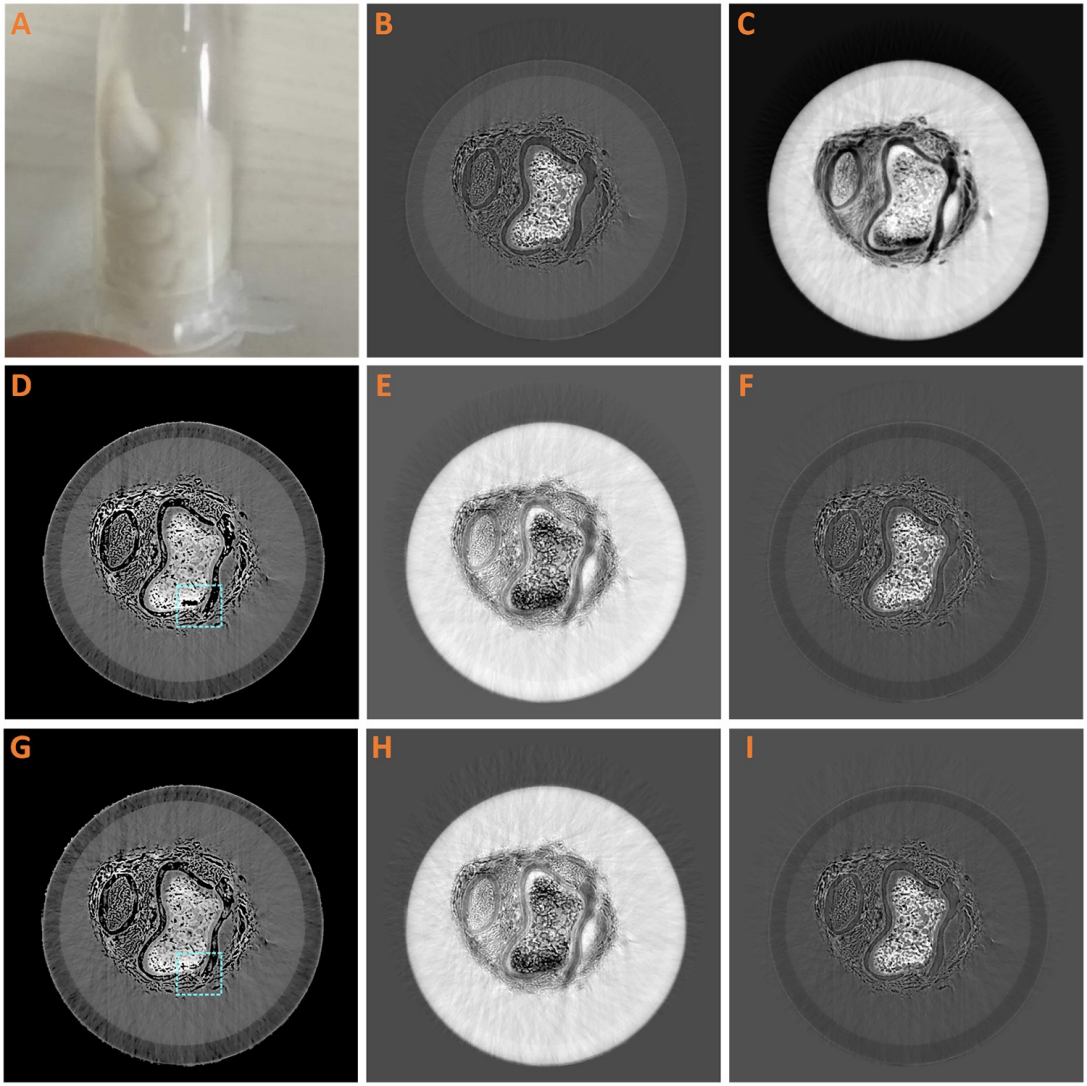
Biological sample result. (A) Chicken paw. The tomogram of ***μ*** (B) and ***δ*** (C). (D) and (G) Equivalent atomic number. (E)(H) Decomposed image of water. (F)(I) Decomposed image of bone. (D-F) are the result of image-based method, (G-I) are result of PAMD-SART. The display window for B is [-0.2, 0.8], C [-0.1, 5.3], D and G [3.3, 16.5], E and H [-0.4, 1.1], F and I [-0.4, 1].

Taking into account the blooming applications in biological sample imaging, we employed a chicken paw bought from supermarket as the specimen. The results are shown in Fig 7. In the second row of Fig 7(D)–7(F) are the result of image-based method. In the third row, (G)-(I) are the result of PAMD-SART. Since the sample is complex, in order to keep the image fidelity, the regularization term at all reconstruction was small.

In this experiment, the refraction image contained more artifacts than the absorption image. The reason lied in the dry chicken paw included a lot of air regions, which caused a sharp change in the refractive index and further led to these artifacts. It was noticeable that the noise of the water-based and bone-based tomographic images was not amplified by the PAMD-SART method, but almost the same as the image-based method. In the equivalent atomic number image, the *Z*_*C*_ of water in PAMD-SART method was more close to theoretical value than the image-based method. As is shown at the dotted frame part of bone in Fig 7D and 7G, the image-based method suffers an error at some part. It is because ***δ*** is less than zero, but ***μ*** larger than zero. However, the PAMD-SART method avoids this error, since it adopts the projection information of both ***μ*** and ***δ*** in the reconstruction process. Thus, it indicates that the proposed PAMD-SART method is also better than image-based method at complex biological applications.

## Discussion and conclusion

In this paper, we propose an iterative method, PAMD-SART, for phase and absorption based substrate decomposition. This method directly reconstructs basic images with desirable noise suppression and boundary maintenance. Numerical simulations and real experiments validate the superiority of the proposed method than the traditional image-based method. In addition, the obtained absorption and phase images are further quantitatively analyzed in terms of equivalent atomic number. The corresponding results are close to the theoretical values, and are more precise than the dual-energy method for low X-ray energies. The proposed PAMD-SART method works well for the synchrotron monochromatic projection data, and will be further extended to the laboratory light source CT system according to the equivalent monochromatic method.

The phase contract CT has not yet been applied to clinical practical applications, and is mainly limited by the manufacture of gratings [28–30]. The method proposed in this paper is a primary research. We verify the effectiveness of PAMD-SART with the data acquired by grating interferometer at synchrotron radiation station. And the size of phantom is just about 10mm due to the limitation of low energy and the limited size of grating and detector.

When calculating the equivalent atomic number (*Z*), there is actually no obvious comparability between phase CT and dual energy CT. It is because the principles of these two imaging patterns are not the same. However, the comparison experiments show that dual energy has more clear conditions for *Z* calculation. In the case of low energy (less than 30 keV), using two absorption coefficient equations which ignore the coherent scattering (Rayleigh scattering) term to solve *Z* will have a large error. While in conventional applications of medical or industrial CT, the photon energy is relatively high (more than 40 keV), so it is feasible to ignore the coherent scattering term. Validation and comparison in polychromatic and high energy X-ray are beyond the scope of this study, and will be investigated in future.

## Supporting information

S1 Data (ZIP)

## References

[1] Lusic H, Grinstaff MW. X-ray-computed tomography contrast agents. Chemical Reviews. 2013;113(3):1641–1666. 10.1021/cr200358s 23210836 PMC3878741

[2] Townsend DW, Carney JPJ, Yap JT, Hall NC. PET/CT today and tomorrow. Journal of Nuclear Medicine. 2004;45 Suppl 1(supplement 1):4S 14736831

[3] Pfeiffer F, Weitkamp T, Bunk O, David C. Phase retrieval and differential phase-contrast imaging with low-brilliance X-ray sources. Nature Physics. 2006;2(4):258–261. 10.1038/nphys265

[4] Huang ZF, Kang KJ, Li Z, Zhu PP, Yuan QX, Huang WX, et al Direct computed tomographic reconstruction for directional-derivative projections of computed tomography of diffraction enhanced imaging. Applied Physics Letters. 2006;89(4):041124 10.1063/1.2219405

[5] Zou Y, Pan X. Exact image reconstruction on PI-lines from minimum data in helical cone-beam CT. Physics in Medicine and Biology. 2004;49(6):941–959. 10.1088/0031-9155/49/6/006 15104318

[6] Sidky EY, Kao CM, Pan X. Accurate image reconstruction from few-views and limited-angle data in divergent-beam CT. Journal of X Ray Science and Technology. 2009;14(2):119–139.

[7] Zhang K, Hong Y, Zhu P, Yuan Q, Huang W, Wang Z, et al Study of OSEM with different subsets in grating-based X-ray differential phase-contrast imaging. Analytical and Bioanalytical Chemistry. 2011;401(3):p.837–844. 10.1007/s00216-011-5080-6 21626196

[8] Wang ZT, Kang KJ, Huang ZF, Chen ZQ. Quantitative grating-based x-ray dark-field computed tomography. Applied Physics Letters. 2009;95(9):094105 10.1063/1.3213557

[9] Qi Z, Zambelli JN, Chen GH. Quantitative imaging of electron density and effective atomic number using phase contrast CT. Physics in Medicine and Biology. 2010;55(9):2669–2677. 10.1088/0031-9155/55/9/016 20400806 PMC3746746

[10] Han HJ, Wang SH, Gao K, Wang ZL, Zhang C, Yang M, et al Preliminary research on dual-energy X-ray phase-contrast imaging. Chinese Physics C. 2016;40(4):048201 10.1088/1674-1137/40/4/048201

[11] Han H, Hu R, Wali F, Wu Z, Gao K, Wang S, et al Phase-contrast imaging for body composition measurement. Physica Medica. 2017;43:25–33. 10.1016/j.ejmp.2017.10.006 29195559

[12] Braig E, Böhm J, Dierolf M, Jud C, Günther B, Mechlem K, et al Direct quantitative material decomposition employing grating-based X-ray phase-contrast CT. Scientific Reports. 2018;8(16394). 10.1038/s41598-018-34809-6 30401876 PMC6219573

[13] Thüring T, Modregger P, Pinzer B, Wang Z, Stampanoni M. Non-linear regularized phase retrieval for unidirectional X-ray differential phase contrast radiography. Optics express. 2011;19:25545–58. 10.1364/OE.19.025545 22273948

[14] Yan W, Huang W, He Q, Zhu Z, Zhu P. Adaptive weighted total variation regularized phase retrieval in differential phase-contrast imaging. Optical Engineering. 2018;57(5):1 10.1117/1.OE.57.11.117114

[15] Pfeiffer F, Bech M, Bunk O, Kraft P, Eikenberry EF, Ch B, et al Hard-X-ray dark-field imaging using a grating interferometer. Nature Materials. 2008;7(2):134–137. 10.1038/nmat2096 18204454

[16] Rudin LI, Osher S, Fatemi E. Nonlinear total variation based noise removal algorithms. Physica D Nonlinear Phenomena;60(1-4):259–268. 10.1016/0167-2789(92)90242-F

[17] Chambolle A. An Algorithm for Total Variation Minimization and Applications. Journal of Mathematical Imaging and Vision. 2004;20(1-2):89–97.

[18] Li X, Lu C, Yi X, Jia J. Image Smoothing via L0 Gradient Minimization. In: the 2011 SIGGRAPH Asia Conference; 2011.

[19] Zhu Y, Wang Q, Li M, Jiang M, Zhang P. Image reconstruction by Mumford-Shah regularization for low-dose CT with multi-GPU acceleration. Physics in Medicine and Biology. 2019;64(15):155017–. 10.1088/1361-6560/ab2c85 31239414

[20] Andersen AH, Kak AC. Simultaneous algebraic reconstruction technique (SART): A superior implementation of the ART algorithm. Utrason Imag. 1984; p. 81–94. 10.1177/016173468400600107 6548059

[21] Z Y, F N, F D, A W, G L, J H. Simulation tools for two-dimensional experiments in x-ray computed tomography using the FORBILD head phantom. Physics in medicine and biology. 2012;57(13):237–252. 10.1088/0031-9155/57/13/N237 PMC3426508 22713335

[22] Río MSD, Dejus RJ. XOP v2.4: recent developments of the x-ray optics software toolkit. Proceedings of SPIE. 2011;8141(5):259–264.

[23] Weitkamp T, Diaz A, David C, Pfeiffer F, Stampanoni M, Cloetens P, et al X-ray phase imaging with a grating interferometer. Optics express. 2005;13:6296–304. 10.1364/OPEX.13.006296 19498642

[24] Spiers F W. Effective Atomic Number and Energy Absorption in Tissues*. British Journal of Radiology. 1946;19(218):52 10.1259/0007-1285-19-218-52 21015391

[25] Tsunoo T, Torikoshi M, Ohno Y, Endo M, Natsuhori M, Kakizaki T, et al. Measurement of electron density and effective atomic number using dual-energy X-ray CT. In: Nuclear Science Symposium Conference Record; 2004.

[26] Schoonjans T, Brunetti A, Golosio B, del Rio MS, Solé VA, Ferrero C, et al The xraylib library for X-ray-matter interactions. Recent developments. Spectrochimica Acta Part B Atomic Spectroscopy;66(11-12):776–784. 10.1016/j.sab.2011.09.011

[27] Plechaty EF, Cullen LE, Howerton RJ. Tables and Graphs of Photon-Interaction Cross-sections from 10 eV to 100 MeV Derived from the LLL Evaluated Nuclear Data Library. Unknown. 1975; 1.

[28] Zan G, Vine DJ, Spink RI, Yun W, Wang Q, Wang G. Design optimization of a periodic microstructured array anode for hard x-ray grating interferometry. Physics in Medicine and Biology. 2019;64(14):145011–. 10.1088/1361-6560/ab26ce 31163408 PMC6850769

[29] Thüring T, Modregger P, Grund T, Kenntner J, David C, Stampanoni M. High resolution, large field of view x-ray differential phase contrast imaging on a compact setup. Applied Physics Letters. 2011;99(4):3287.

[30] Revol V, Kottler C, Kaufmann R, Jerjen I, Lüthi T, Cardot F, et al X-ray interferometer with bent gratings: Towards larger fields of view. Nuclear Instruments and Methods in Physics Research. 2011;648(supp-S1):S302–S305. 10.1016/j.nima.2010.11.040

